# Aloperine Protects Mice against Bleomycin-induced Pulmonary Fibrosis by Attenuating Fibroblast Proliferation and Differentiation

**DOI:** 10.1038/s41598-018-24565-y

**Published:** 2018-04-19

**Authors:** Wanling Yin, Jing Han, Zhijun Zhang, Zaomu Han, Siyuan Wang

**Affiliations:** 1 Department of Gerontology, The Central Hospital of Wuhan, Tongji Medical College, Huazhong University of Science and Technology, Wuhan, China; 2 Department of oncology, Tongji Hospital, Tongji Medical College, Huazhong University of Science and Technology, Wuhan, China; 3 Reproductive medicine center, Taihe Hospital, Hubei University of Medicine, Shiyan, China

## Abstract

Aloperine is a quinolizidine alkaloid extracted from Sophora alopecuroides. It has been proven to alleviate oxidative stress and effectively promote tumor cell apoptosis in mice. Herein, we investigated whether aloperine could also mediate its protective effects on bleomycin (BLM)-induced pulmonary fibrosis. Pathological staining, western blot, RT-PCR and flow cytometry were used to evaluate the impact of aloperine on the development of pulmonary fibrosis. The effect of aloperine on fibroblast proliferation, differentiation and related signaling pathways were next investigated to demonstrate the underlying mechanisms. In the present report, we showed that aloperine provided protection for mice against BLM-induced pulmonary fibrosis as manifested by the attenuated lung injury and reduced fibrosis along with alleviated fibroblast proliferation and differentiation. Additionally, we provided *in vitro* evidence revealing that aloperine inhibited cellular proliferation in PDGF-BB-stimulated mouse lung fibroblasts by repressed PI3K/AKT/mTOR signaling and fibroblast to myofibroblast differentiation by repressed TGF-β/Smad signaling. Overall, our data showed that aloperine could protect the mice against BLM-induced pulmonary fibrosis by attenuated fibroblast proliferation and differentiation, which indicated that aloperine may be therapeutically beneficial for IPF patients.

## Introduction

Idiopathic pulmonary fibrosis (IPF) is a chronic, progressive, and devastating lung disease with unknown etiology and manifested the poorest prognosis^1^. Despite past extensive studies, the underlying mechanisms of IPF pathogenesis are not fully understood^2^. As a result, therapeutic strategies for IPF have been largely unsuccessful, and the average survival time for this category of patients is only 3 to 5 years after diagnosis^2^.

The pathology of IPF is characterized by migration, proliferation and differentiation of fibroblast and remodeling of the extracellular matrix (ECM)^3^. Fibroblasts have been noted to play a central role in the fibrotic processes regulated by transforming growth factor-β (TGF-β)^4,5^ or other profibrotic mediators, such as platelet-derived growth factor (PDGF)^6,7^ and connective tissue growth factor (CTGF)^8^. These fibroblasts characterized by abnormal α-SMA and fibrillar collagens expression are called myofibroblasts^9^. Fibroblast to myofibroblast differentiation is a key step during the course of fibrotic process^10^. It has been shown that myofibroblasts in IPF lung tissues exhibited a profibrotic secretory phenotype, with aberrantly proliferative rates and lower spontaneous apoptosis^11^. To reduce the fibrogenesis in IPF, the production of these profibrotic mediators and myofibroblast differentiation must be attenuated^12–15^.

Aloperine is a kind of alkaloid extracted from sophora alopecuroides and has been reported to execute therapeutic effects against pulmonary hypertension^16^, renal injury^17^ and neuropathic pain^18^ through attenuating oxidative stress, and multiple myeloma^19^, and colon cancer^20^ though increasing cell apoptosis. These observations prompted us to hypothesize that aloperine may be a good candidate drug for the prevention and treatment of bleomycin (BLM) -induced pulmonary fibrosis, since oxidative stress and apoptosis are involved in its pathogenesis^21,22^. To address this feasibility, we conducted studies in a pulmonary fibrosis mouse model, and then assessed the impact of aloperine on the disease development. We found that administration of aloperine provided protection for mice against pulmonary fibrosis as manifested by the attenuated lung injury and reduced fibrosis along with a marked alleviation of fibroblast proliferation and differentiation in the lung. Mechanistic studies revealed that aloperine could regulate the phosphatidylinositol-3-kinase/protein kinase B/mammalian target of rapamycin (PI3K/AKT/mTOR) and TGF-β/Smad signaling pathways, and by which it reduced fibroblast proliferation and differentiation, respectively. Our data suggested that treatment with aloperine could be a viable strategy for the prevention and treatment of pulmonary fibrosis in clinical settings.

## Results

### Aloperine administration ameliorated BLM-induced lung injury and fibrosis

**Figure 1:**
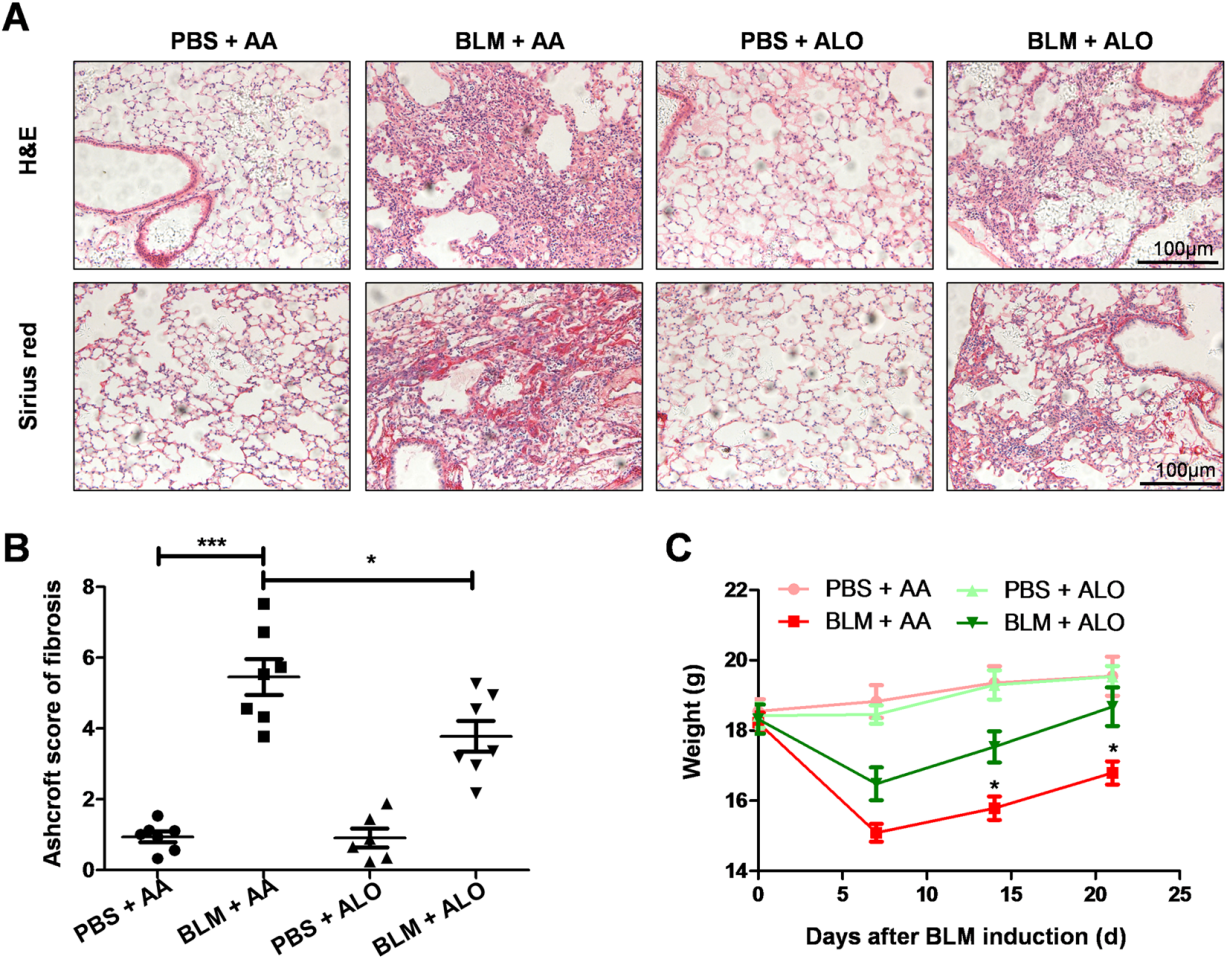
Histological analysis of the effects of aloperine on the severity of lung injury and fibrosis after BLM induction. (**A**) Representative results for H&E (Up) and Sirius red (Down). Images were taken under × 200 amplification. (**B**) Quantitative mean Ashcroft scores for fibrosis. (**C**) Body weight change during the course of pulmonary fibrosis. Six to 7 mice were included in each study group. Statistical analysis was performed by one-way ANOVA with Newman-Keuls post-hoc test. *p < 0.05; ***p < 0.001.

Aloperine is known to treat various diseases. However, whether it could be used as a viable approach for BLM-induced pulmonary fibrosis has not been extensively researched. To study the effect of aloperine on BLM-induced pulmonary fibrosis, the mice were treated with aloperine for 21 days after exposure to BLM. Mice treated with phosphate-buffered saline (PBS) + Alo or PBS + AA served as controls. We first sought to address the impact of aloperine on pulmonary fibrosis. A significantly attenuated lung injury and pulmonary fibrosis were noted in aloperine-treated mice as evidenced by hematoxylin and eosin (H&E) and Sirius red staining (Fig. 1A). Particularly, the severity of pulmonary fibrosis was much lower as manifested by the lower Ashcroft scores (5.45 ± 0.51 versus 3.78 ± 0.43, p < 0.05; Fig. 1B), while mice originating from the PBS + Alo group manifested similar levels of fibrotic scores to those of mice derived from the PBS + AA group. In addition, mice derived from the aloperine or vehicle group exhibited significant weight loss on day 7 after BLM induction compared to the control group. Of note, aloperine treatment after BLM challenge resulted in less weight loss in comparison to the BLM group treated with vehicle on day 14 and 21 (Fig. 1C).

**Figure 2:**
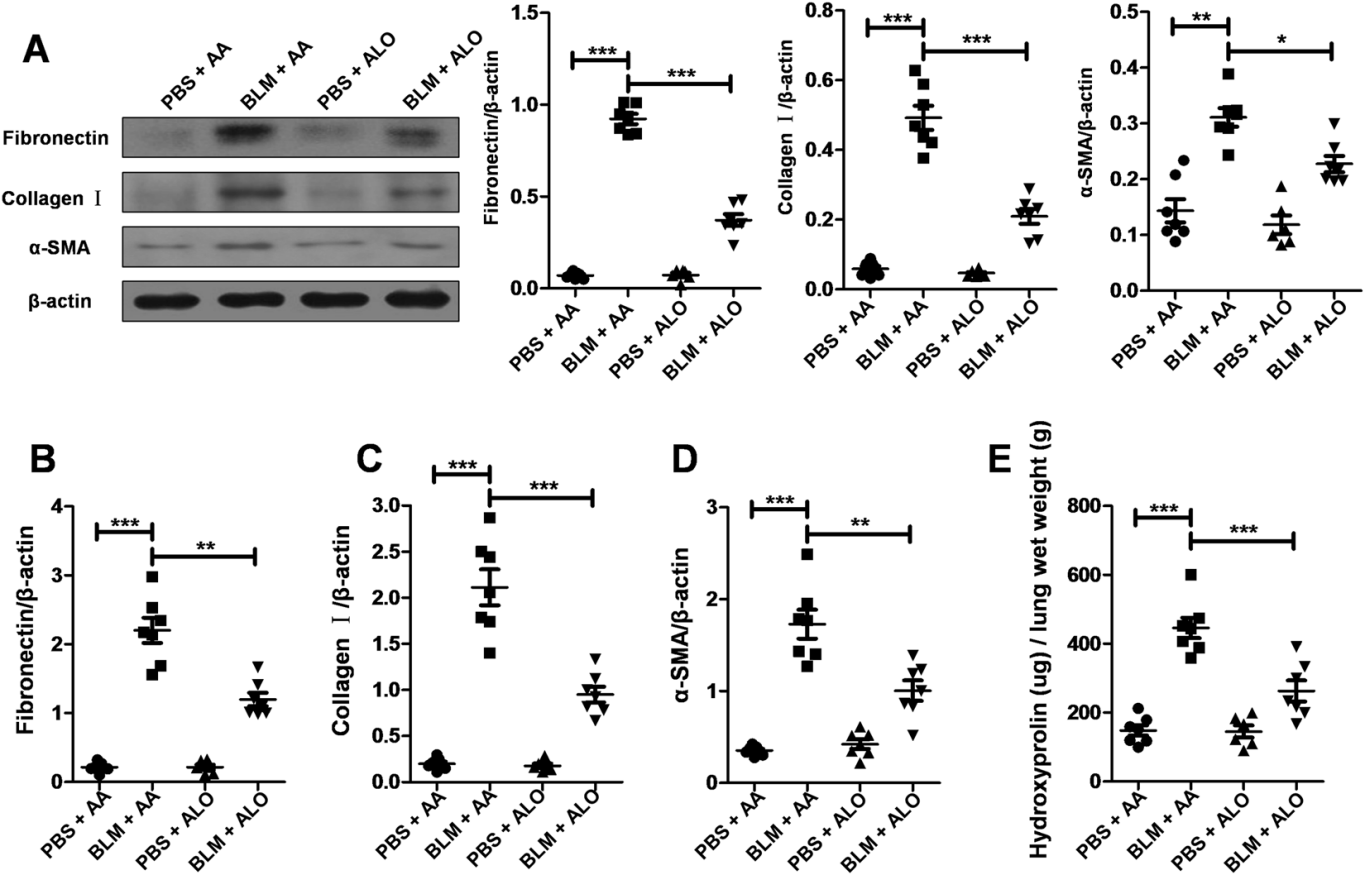
Administration of aloperine attenuated lung fibrosis after BLM induction. (**A**) Analysis of fibrotic markers in lung homogenates. Left panel: representative western blot results for fibronectin, collagen I, and α-SMA expression. Right panel: graphic figures showing the western blot results of all mice examined. (**B**–**D**) RT-PCR analysis of fibronectin, collagen I, and α-SMA expression in the lung induced by BLM. (**E**) The rates of hydroxyproline and lung wet weight of all mice studied. Six to 7 mice were included in each study group. Statistical analysis was performed by one-way ANOVA with Newman-Keuls post-hoc test. *p < 0.05; **p < 0.01; ***p < 0.001.

To further evaluate the effects of aloperine on pulmonary fibrosis, we examined the levels of fibronectin, collagen I, and α-smooth muscle actin (α-SMA) in the lung homogenates by western blot. As expected, the expression of fibronectin, collagen I, and α-SMA were increased in the group induced by BLM compared with the PBS-treated control mice. Notably, mice that originated from the BLM + Alo group exhibited a marked reduction of fibrotic markers compared with BLM + vehicle group mice (Fig. 2A). Similar results were observed by reverse transcription-polymerase chain reaction (RT-PCR) analysis of fibronectin, collagen I, and α-SMA expression (Fig. 2B–D). To confirm these observations, we examined the content of hydroxyproline in the lung. BLM caused higher hydroxyproline levels in the lung tissue. However, the administration of aloperine displayed low levels of hydroxyproline after BLM induction (Fig. 2E).

Since aloperine is known for reducing inflammation, we next sought to determine the impact of aloperine on the lung inflammation. As expected, severe inflammatory responses were observed in the BLM + AA group after 8 days of BLM exposure, as demonstrated by the infiltration of inflammatory cells into the lung (Supplementary Figure 1A). Next, we compared the number and subtype of inflammatory cells in the bronchoalveolar lavage fluid (BALF). The total number of inflammatory cells in the BALF was significantly reduced in the BLM + Alo group compared with the BLM + AA group (Supplementary Figure 1B). Specifically, BALF derived from BLM + Alo group contained significantly fewer macrophages (Supplementary Figure 1C), lymphocytes (Supplementary Figure 1D) compared with the BLM + AA group. However, the total number of neutrophils was not significantly different between the BLM + Alo and BLM + AA groups (Supplementary Figure 1E).

Together, these data demonstrated that administration of aloperine provided protection for mice against BLM-induced lung injury and fibrosis.

### Aloperine treatment suppressed reactive oxygen species (ROS) production in the lungs of mice induced by BLM

**Figure 3:**
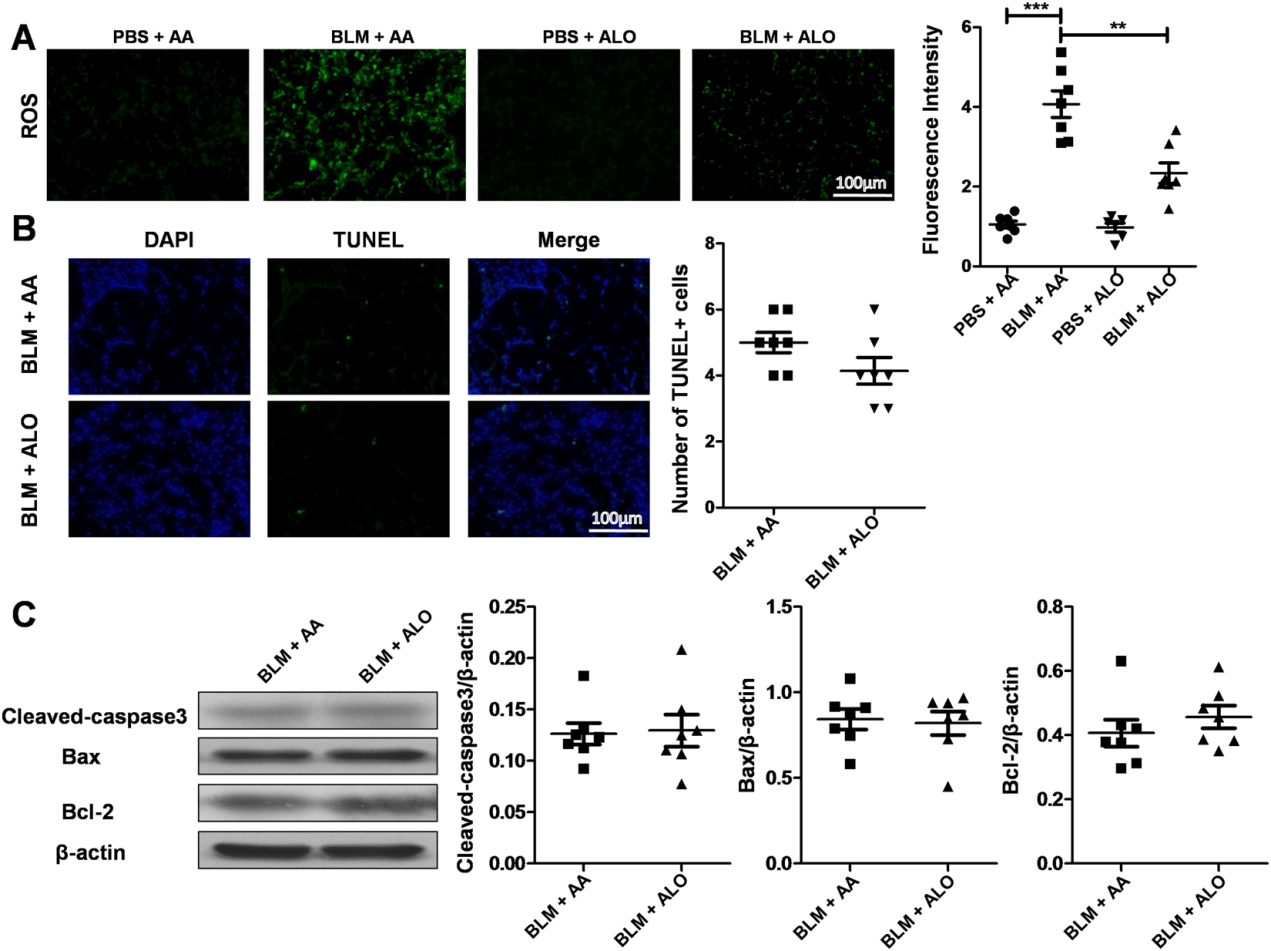
Analysis of ROS accumulation and apoptosis following BLM induction. (**A**) Analysis of ROS production. Left panel: representative results for detection of DCFH-DA fluorescence in lung sections. Right panel: quantitative mean fluorescence intensity of all mice studied. (**B**) Analysis of apoptosis. Left panel: Representative images for TUNEL assays of lung sections. Right panel: Quantitative analysis of TUNEL-positive cells. Images were taken under ×200 amplification. (**C**) Analysis of apoptosis-related proteins in lung homogenates. Left panel: representative western blot results for cleaved-caspase3, Bax, and Bcl-2 expression. Right panel: graphic figures showing the western blot results. Six to 7 mice were included in each study group. Statistical analysis was performed by one-way ANOVA with Newman-Keuls post-hoc test or Mann-Whitney test. **p < 0.01; ***p < 0.001.

Previous studies have shown that aloperine could be used as an effective candidate for pulmonary hypertension, renal injury, and neuropathic pain by inhibiting oxidative stress^16–18^. Given that oxidative stress plays an important role in the pathogenesis of pulmonary fibrosis by promoting epithelial cell apoptosis^22^, ROS production was investigated by staining with dichloro-dihydro-fluorescein diacetate (DCFH-DA) in the lung sections. Similar to previous results, a significant ROS accumulation was found in the mice from the BLM + AA group compared with that of mice from the PBS + AA group, while aloperine administration led to a 50% reduction of ROS accumulation (Fig. 3A). Furthermore, a similar result was observed for the levels of ROS in cultured mouse lung fibroblasts (Supplementary Figure 2). We next sought to detect the cell apoptosis by TUNEL staining in the lung sections. However, unlike its impact on ROS accumulation, aloperine treatment did not seem to affect the cell apoptosis, as we failed to detect perceptible differences in the number of TUNEL-positive cells (Fig. 3B). Consistently, the lung samples derived from the BLM + AA and BLM + Alo groups exhibited comparable levels of cleaved-caspase3, Bax and Bcl-2 (Fig. 3C).

### Administration of aloperine inhibited fibroblasts proliferation

**Figure 4:**
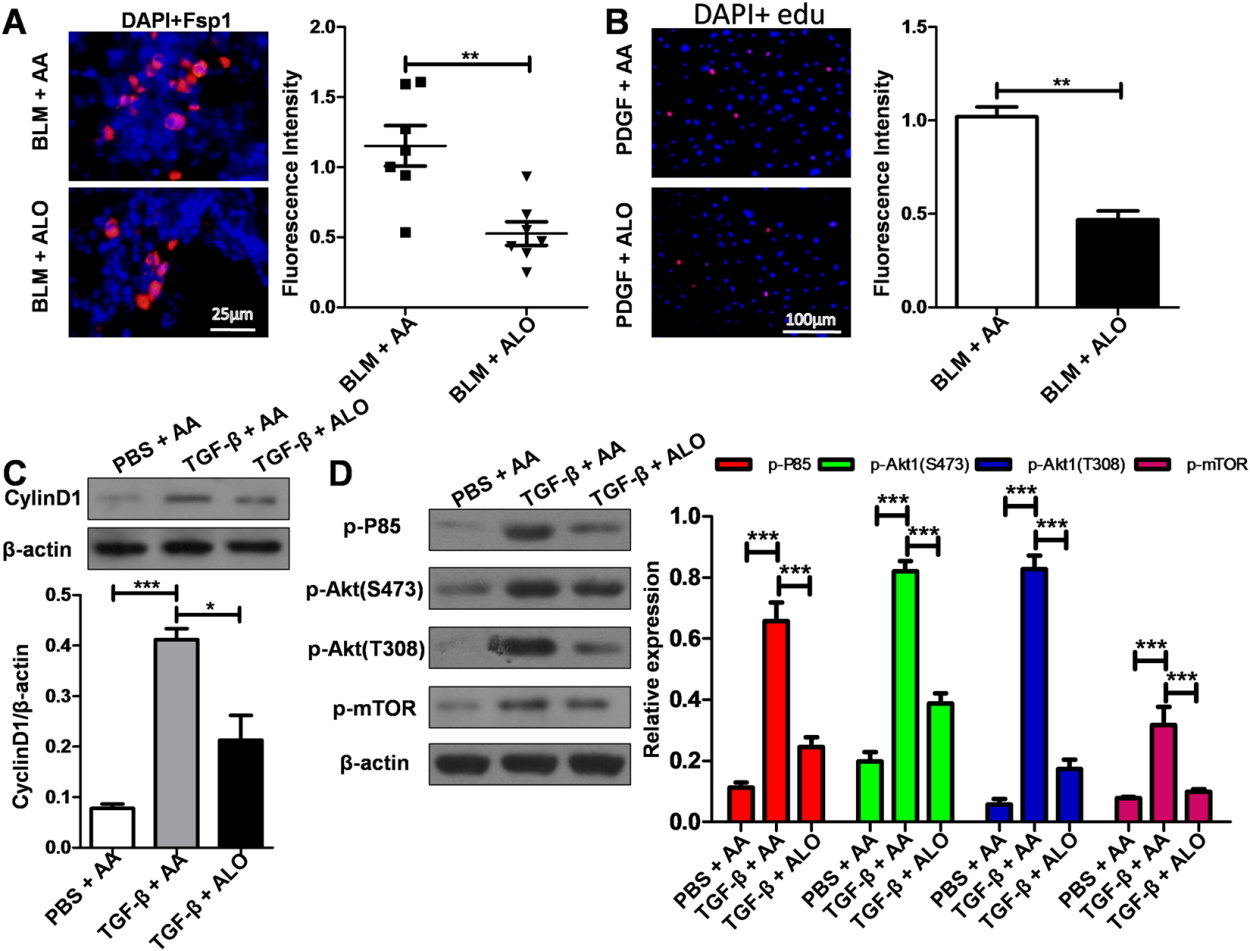
Aloperine treatment suppresses fibroblast proliferation. (**A**) Results for Fsp1 expression in the lung sections. Left panel: representative immunostaining results of Fsp1. Right panel: bar graphic figure of Fsp1 for all mice examined. (**B**) Analysis of lung fibroblast proliferation after PDGF-BB stimulation. Left panel: representative EdU staining. Right panel: A bar graphic figure of EdU fluorescence intensity. (**C**) Analysis of cyclin D1expression. Left panel: representative western blot results for cyclin D1 expression. Right panel: graphic figures showing the western blot results. (**D**) The impact of aloperine on PDGF-BB stimulated PI3K/AKT/mTOR signaling in mouse lung fibroblasts. Left panel: representative western blot results for the levels of p-P85, p-AKT (Ser473 and Thr308), and p-mTOR. Right panel: graphic figures showing the western blot results with 3 replications. Statistical analysis was performed by Two-way ANOVA with Newman-Keuls post-hoc test or Mann-Whitney test. *p < 0.05; **p < 0.01; ***p < 0.001.

The proliferation of fibroblasts has been suggested to be one of major pathophysiological components of pulmonary fibrosis^23^. We therefore conducted immunostaining to examine the number of fibroblasts in the lungs of mice. Indeed, compared with the BLM + Alo group, more fibroblasts were observed in the lungs of mice from the BLM + AA group, as evidenced by high levels of fibroblast-specific protein 1 (Fsp1) expression, a marker of mouse lung fibroblasts^24^ (Fig. 4A).

To confirm the above observations, we then assessed the impact of aloperine on lung fibroblast proliferation induced by PDGF-BB for 48 h. Indeed, EdU staining analysis revealed that PDGF-BB significantly stimulated lung fibroblast proliferation, which was repressed by aloperine (Fig. 4B). Consistently, PDGF-BB induced high levels of cyclin D1 expression, while cyclin D1 was significantly low upon the addition of aloperine (Fig. 4C).

It has been suggested that PI3K/AKT/mTOR signaling is critical for the proliferation of fibroblasts upon PDGF-BB stimulation^25^. We therefore examined the impact of aloperine on PI3K/AKT/mTOR signaling in mouse lung fibroblasts stimulated with PDGF-BB for 3h. An increase in the p-P85 levels was detected after 3 h of PDGF-BB stimulation, while the levels of p-P85 were significantly low when treated with aloperine (Fig. 4D). Furthermore, a similar trend was observed for the levels of p-AKT (Ser473 and Thr308) and p-mTOR (Fig. 4D). Taken together, our results indicate that aloperine suppressed the proliferation of fibroblasts by repression of PI3K/AKT/mTOR signaling.

### Aloperine treatment suppressed the differentiation of fibroblasts

**Figure 5:**
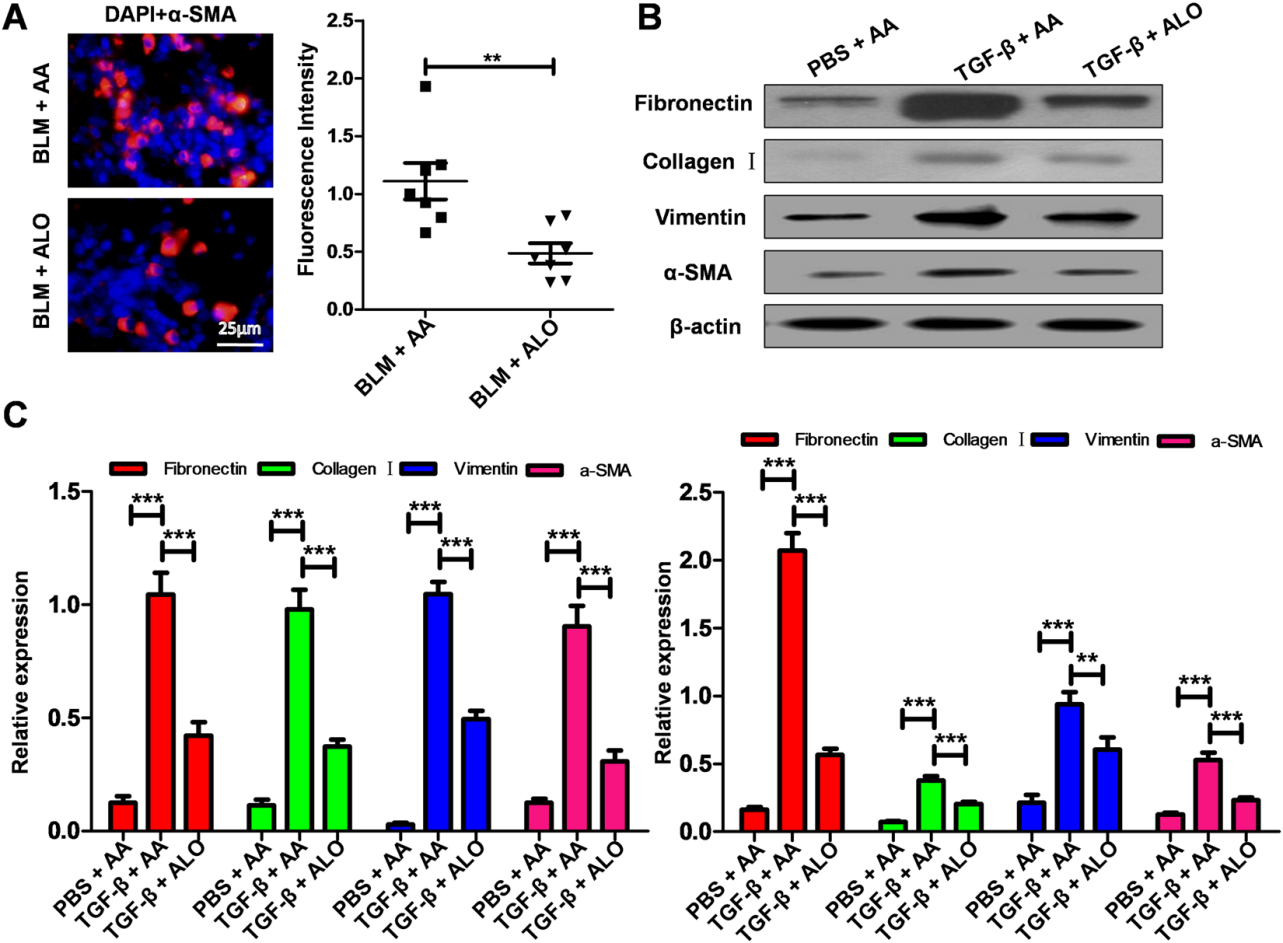
Aloperine treatment inhibited fibroblast differentiation. (**A**) Immunostaining of α-SMA expression in BLM-induced lung sections. Left panel: representative images for α-SMA expression. Right panel: quantitative mean fluorescence intensity of all mice studied. (**B**) Analysis of lung fibroblast differentiation after TGF-β stimulation. Up panel: representative western blot results for fibronectin, collagen I, vimentin, and α-SMA. Down panel: bar graphic figure for western blot results. (**C**) Analysis of lung fibroblast differentiation by RT-PCR following TGF-β. Statistical analysis was performed by Two-way ANOVA with Newman-Keuls post-hoc test or Mann-Whitney test. **P < 0.01; and ***P < 0.001.

Because fibroblast differentiation was a key step during the course of the fibrotic process, we next assessed the impact of aloperine on the differentiation of fibroblast in the lungs of the mice from the BLM + AA and BLM + Alo group. Similar to previous results (Fig. 2A,D). Immunostaining showed that there were more α-SMA positive cells in mice from the BLM + AA group compared with the BLM + aloperine group (Fig. 5A), indicating that administration of aloperine may affect the differentiation of fibroblasts.

Based on the above observations, we next utilized mouse lung fibroblasts to validate the effects of aloperine on fibroblasts differentiation. Interestingly, administration of aloperine significantly inhibited fibroblasts differentiation as evidenced by the significantly reduced levels of fibronectin, collagen I, vimentin, and α-SMA after TGF-β treatment for 24 h analyzed by western blot (Fig. 5B) and RT-PCR (Fig. 5C).

### Aloperine attenuated fibroblast differentiation by suppression of TGF-β/Smad signaling

**Figure 6:**
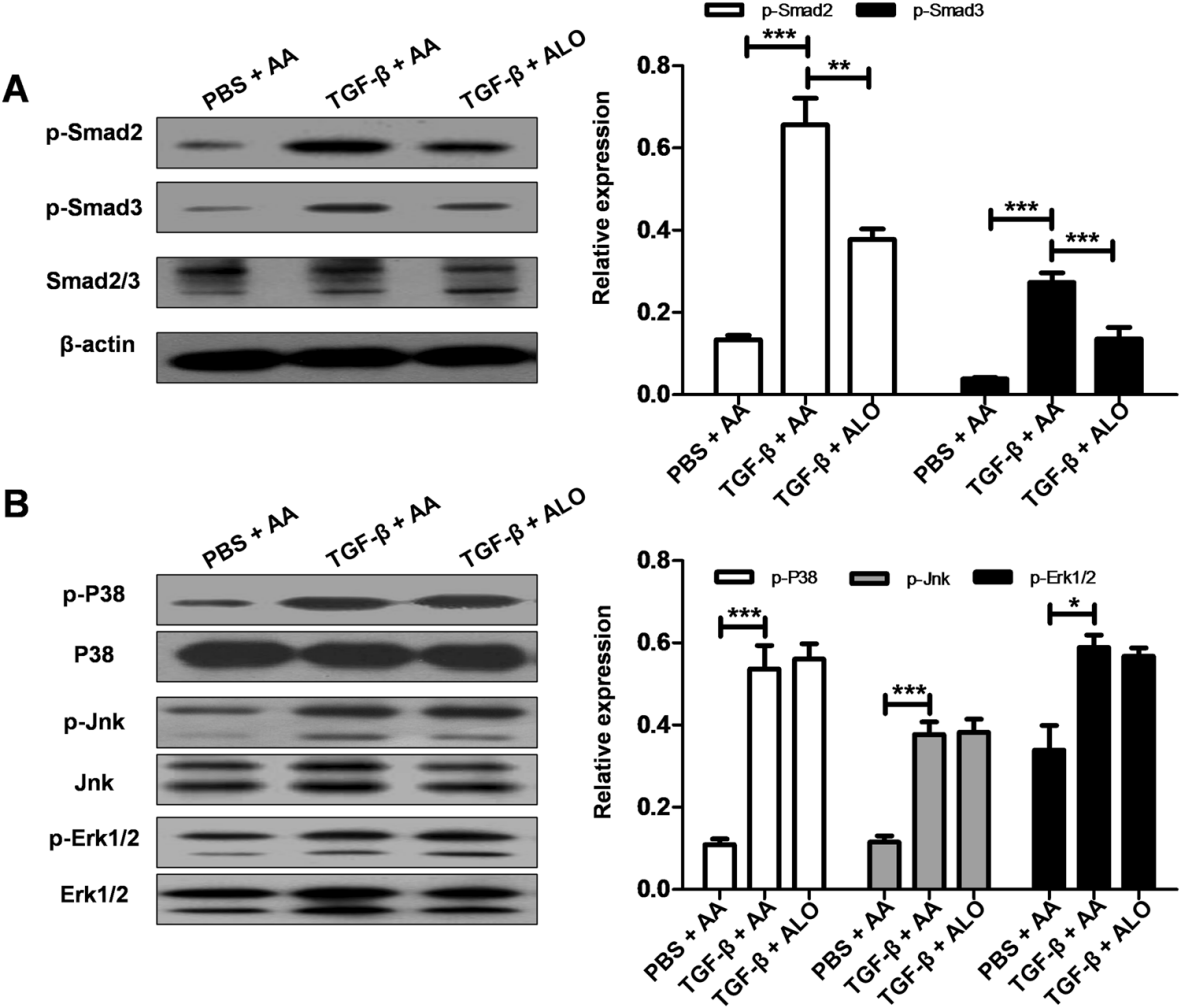
The impact of aloperine on TGF-β-stimulated Smad signaling in mouse lung fibroblasts. (**A**) Administration of aloperine attenuated TGF-β-induced Smad signaling. Left panel: representative western blot results for p-Smad2, p-Smad3, and Smad2/3 at 3 h after TGF-β stimulation. Right panel: bar graphic figure showing the data with 3 replications. (**B**) Aloperine treatment did not affect TGF-β-induced MAPK signaling. Left panel: representative western blot results for p-P38/P38, p-Jnk/Jnk, p-Erk1/2/Erk1/2 at 3 hafter TGF-β stimulation. Right panel: bar graphic figure showing the data with 3 replications. Statistical analysis was performed by Two-way ANOVA with Newman-Keuls post-hoc test. **p < 0.01; ***p < 0.001.

The above results suggested that aloperine could alleviate the differentiation of fibroblasts. To gain insight into the mechanisms underlying aloperine inhibition of the differentiation of fibroblasts, we examined the activities of the Smad signaling pathway, which was critical for optimal and sustained fibroblast differentiation upon TGF-β stimulation. Indeed, TGF-β stimulation for 3 h significantly induced Smad signal activation as manifested by increasing levels of p-Smad2 and p-Smad3, while aloperine treatment significantly attenuated Smad signal activation (Fig. 6A). Additionally, MAPK signaling was also implicated in TGF-β-induced fibroblast differentiation^20^. However, we failed to detect a significant difference in terms of the phosphorylated forms of p38, JNK, and ERK1/2 between the two groups (Fig. 6B). Collectively, our data supported the hypothesis that administration of aloperine attenuated the differentiation of fibroblasts by repressed TGF-β/Smad signaling

## Discussion

In the present report, we conducted studies *in vivo* and *in vitro* to determine the therapeutic potential for aloperine in pulmonary fibrosis. We have provided convincing evidence suggesting that aloperine treatment effectively decreases lung injury and pulmonary fibrosis and alleviates fibroblast proliferation and differentiation in mice with BLM-induced pulmonary fibrosis. The underlying mechanisms of the protective effects of aloperine might be ascribed to inhibition of the PI3K/AKT/mTOR and TGF-β/Smad signaling pathways. These data might suggest potential clinical applications of aloperine in the treatment of pulmonary fibrosis in clinical settings.

Accumulating evidence has indicated that aloperine had anti-inflammatory and anti-tumor therapeutic functions in animal studies^16–20,26,27^. The mechanistic studies of aloperine have primarily focused on its anti-oxidant, anti-inflammatory and apoptosis-promoting properties. The production of ROS has been noted to be essential to the development of pulmonary fibrosis by regulating the apoptosis of lung epithelial cells^22^. In line with previous results, aloperine treatment indeed decreased the ROS production in the lung induced by BLM as manifested by the reduction of DCFH-DA fluorescence intensity in lung sections, which suggests that aloperine may have an anti-oxidant role in the progression of pulmonary fibrosis. However, we failed to detect perceptible differences in the number of TUNEL-positive cells in the lung, which indicated that the protective roles of aloperine on pulmonary fibrosis did not seem to depend on reducing the cell apoptosis induced by ROS.

Fibroblasts were found to play a central role in the fibrotic process and contributed to histological features of IPF lung tissues because fibroblasts were accompanied by an increased intracellular ROS generation in IPF patients compared with the healthy controls, and ROS was an essential mediator of Smad2/3 transcription factor activation in response to TGF-β in fibroblasts^28^. We thus detected the impact of aloperine on fibroblast differentiation *in vivo* and *in vitro*. As expected, BLM caused an increase of fibroblast-to-myofibroblast differentiation in the lungs as evidenced by the increasing number of α-SMA positive cells. Interestingly, administration of aloperine significantly inhibited the fibroblast differentiation. To further confirm these results, we cultured mouse lung fibroblasts and then stimulated them with TGF-β. Surprisingly, aloperine treatment also suppressed fibroblast differentiation.

Accumulation and persistence of fibroblast differentiation are believed to contribute to the development of pulmonary fibrosis. However, the underlying mechanisms of aloperine’s suppression of the fibroblast differentiation have not yet been fully elucidated. Previous studies have revealed that TGF-β can stimulate Smad2 and Smad3 phosphorylation, which directly induces fibroblast differentiation. These observations prompted us to focus on the impact of aloperine on TGF-β/Smad signaling pathway. Indeed, Smad signaling was intensified following TGF-β stimulation in mouse lung fibroblasts. Surprisingly, aloperine treatment markedly inhibited TGF-β-induced Smad2 and Smad3 phosphorylation as evidenced by significantly lower levels of p-Smad2 and p-Smad3 in TGF-β + aloperine treatment mouse lung fibroblasts. Several other signaling pathways have also been found to be closely related to this accumulation and persistence of fibroblast differentiation stimulated by TGF-β, such as the MAPK signaling pathway. However, it seemed that MAPK signaling was not involved in aloperine-mediated fibroblast differentiation as we failed to detect a significant difference in terms of the increase in the phosphorylated forms of p38, JNK, and ERK1/2 with or without aloperine treatment induced by TGF-β. Taken together, these results suggested that aloperine ameliorated fibroblast differentiation at least by inhibition of TGF-β/Smad signaling.

We further assessed the effects of aloperine on fibroblast proliferation, which is a key pathologic feature of IPF. In this study, we demonstrated that mice administered with aloperine following BLM treatment, in part, showed reduced fibroblast proliferation in the fibroblastic foci, as evidenced by low levels of Fsp1 expression. Previous studies have shown that PDGF-BB is a well-described inducer of fibroblast proliferation^29^. Therefore, we assessed the impact of aloperine on PDGF-BB-induced mouse lung fibroblasts proliferation. Indeed, aloperine could significantly repress the proliferation of mouse lung fibroblasts induced by PDGF-BB. Furthermore, cyclin D1, a key protein required for progression through the G1 phase of the cell cycle^29^, was significantly decreased upon the addition of aloperine.

The next critical issue was to dissect the pathways relevant to aloperine’s repression of the proliferation of mouse lung fibroblasts induced by PDGF-BB. Previous studies suggested feasible evidence that PI3K/AKT/mTOR signaling is critical for the proliferation of fibroblasts^30^. To address whether aloperine attenuated the proliferation of mouse lung fibroblasts by repressed PI3K/AKT/mTOR signaling, we first assessed the effect of aloperine on PDGF-BB-induced PI3K activation in mouse lung fibroblasts. Indeed, aloperine administration markedly inhibited PDGF-BB-induced PI3K activation, and consistently, the levels of PI3K downstream signaling, p-AKT, and p-mTOR, were also significantly reduced following PDGF-BB stimulation.

Taken together, our data show that the administration of aloperine protected mice from BLM-induced lung injury and fibrosis. Mechanistic studies have revealed aloperine had a therapeutic function for pulmonary fibrosis by attenuating fibroblast proliferation and differentiation. Specifically, administration of aloperine repressed PI3K/AKT/mTOR and TGF-β/Smad signaling, and thereby attenuated the fibroblast proliferation and differentiation, respectively. However, additional research is needed to determine whether aloperine treatment can reverse the progression of pulmonary fibrosis in established pulmonary fibrosis models to support the potential clinical value of this agent.

## Materials and Methods

### Reagents and Antibodies

BLM was purchased from Nippon Kayaku Co., Ltd. (Japan), while recombinant TGF-β, Fsp1, cleaved-caspase 3, p-Smad2, p-Smad3, and Smad2/3 antibodies were obtained from Cell Signaling (USA). Collagen I, fibronectin, vimentin, α-SMA, and β-actin antibodies were originated from Santa Cruz Biotechnology (USA). Bax, Bcl-2, p-P85, p-AKT (Ser473 and Thr308), p- mTOR, and cyclin D1 were obtained from BD Bioscience (USA).

### Animals

Male C57BL/6 mice (8 weeks old) were obtained from Beijing Huafukang Biology Technology Co., Ltd (Beijing, China) and housed in a specific pathogen-free (SPF) animal facility (12/12 h light/dark cycle) at the Tongji Medical College with sterile acidified water and irradiated food.

All mice in the study were maintained and used according to the protocols approved by the Animal Care and Use Committee at the Central Hospital of Wuhan.

### Animal treatment

All the animals were randomly divided into four groups: (1) BLM-10% acetic acid group (BLM + AA) mice were anesthetized with 1% pentobarbital sodium and then subjected to administration of 0.5 mg/kg of BLM (Nippon Kayaku Co., Ltd., Tokyo, Japan) in sterile PBS intratracheally, and then the mice were treated with PBS containing 10% acetic acid by gavage for 21 consecutive days; (2) BLM-aloperine group (BLM + Alo) mice were treated with 40 mg/kg of aloperine (Santa Cruz, CA, USA) dissolved in PBS containing 10% acetic acid by gavage for 21 consecutive days following BLM injection; (3) PBS-10% acetic acid group (PBS + AA)mice administered with same volume of sterile PBS containing 10% acetic acid served as controls; and (4) PBS-aloperine group (PBS + Alo) control mice were administered with same dose of aloperine orally as described above. All the mice were sacrificed 21 days after BLM administration.

### Histological and immunohistochemical analysis

The left lung was inflated with 4% neutral buffered paraformaldehyde, and the left lung was subsequently removed and placed in fresh 4% neutral buffered paraformaldehyde for 24 h at room temperature. After embedding the tissues in paraffin, they were sliced into 4-µm sections and subjected to H&E and Sirius red staining. Twenty sequential fields of view encompassing the entire lung section were independently and blindly scored by two pathologists with Ashcroft scores as previously described^2^. For immunostaining, fresh frozen sections (6 μm) were co-incubated with primary antibodies against α-SMA (1:200) or Fsp1(1:100), followed by probing with Alexa 594-labeled secondary antibodies (Invitrogen, Carlsbad, CA, USA). The results were assessed by two pathologists using a fluorescent microscope (Olympus, Japan) in a blinded fashion.

### Hydroxyproline content

The hydroxyproline content in the lungs was determined by the commercial kits from Nanjing Jiancheng Bioengineering Institute (Nanjing, China) according to the instructions.

### Assays for ROS accumulation

The left lung was frozen in optimal cutting temperature (OCT) compound immediately without fixation after the mice were sacrificed. Then, the fresh frozen sections (8 μm) were incubated with 2′7′–dichlorodihydrofluorescein diacetate (DCFH-DA, 10 μM, YIJI, Shanghai, China) at 37 °C for 25 min. The sections were immediately subjected to fluorescence analysis under a fluorescence microscope.The fluorescence intensity was quantified by Image-Pro Plus (version 6.0, Datacell) imaging software.

### Culture and treatment of primary mouse lung fibroblasts

Primary mouse lung fibroblasts were obtained from the mouse lung as previously reported^24^. Cells were cultured at 37 °C and 5% CO2 in DMEM supplemented with 10% fetal bovine serum (FBS) and penicillin/streptomycin. The medium was replaced every 3 days. For fibroblast proliferation, the cells were co-cultured with recombinant PDGF-BB (20 ng/ml) + acetic acid or PDGF-BB (20 ng/ml) + aloperine (0.3125 mM) or PBS + acetic acid for the indicated time for protein analysis. For fibroblast differentiation, the cells were co-cultured with recombinant TGF-β (10 ng/ml) + acetic acid,TGF-β (10 ng/ml) + aloperine (0.3125 mM), or PBS + acetic acid for the indicated time for RNA and protein analysis.

### Proliferation assay

Primary lung fibroblast proliferation assay was performed by EdU (5-ethynyl-2′-deoxyuridine) staining according to protocols as reported^31^. Briefly, the mouse lung fibroblasts were treated with recombinant PDGF-BB (20 ng/ml) + acetic acid,PDGF-BB (20 ng/ml) + aloperine (0.3125 mM), or PBS + acetic acid for 48 h. Next, the cells were washed with PBS followed by incubation in serum-free DMEM containing 10 μmol/L EdU (RiboBio, China) for 2 h. Cells were fixed, and then underwent Apollo staining and DNA staining, according to the manufacturer’s instructions.

### Western blot analysis

Lung tissues and cultured cells were homogenized in RIPA lysis buffer (Biyuntian, China). Usually, 20 μg of total proteins per lane were separated by SDS-PAGE and transferred to PVDF membranes, and then the PVDF membranes were incubated with primary antibodies using the established techniques^32^.

### Quantitative RT–PCR analysis

Quantitative RT–PCR analysis was performed using the SYBR Premix Ex Taq (Takara) as reported^33^. The relative expression levels for each target gene were normalized by β-actin. Primers are listed in Table 1.

**Table 1:** The primers used for RT-PCR.

Fibronectin	5′-ATG CAA CGA TCA GGA CAC AA-3′
	5′-TGT GCC TCT CAC ACT TCC AC-3′
Collagen I	5′-CCT GGT AAA GAT GGT GCC-3′
	5′-CAC CAG GTT CAC CTT TCG CAC C-3′
Vimentin	5′-CGG CTG CGA GAG AAA TTG C-3′
	5′-CCA CTT TCC GTT CAA GGT CAA G-3′
β-actin	5′-GTT GTC GAC GAC GAG CG-3′
	5′-GCA CAG AGC CTC GCC TT-3′

### Statistical analysis

All results are expressed as mean ± standard error of mean (SEM) and analyzed with the GraphPad Prism 5.0 software. All data were analyzed by one-way or two-way ANOVA with multiple comparisons or Newman-Keuls-Student t-test where appropriate. In all cases, P < 0.05 was considered statistically significant.

## Electronic supplementary material

Supplementary Figure
