# Supplementary material for: Aloperine Protects Mice against Bleomycin-induced Pulmonary Fibrosis by Attenuating Fibroblast Proliferation and Differentiation
Source: Sci Rep. 2018 Apr 19;8:6265. doi: 10.1038/s41598-018-24565-y (PMC5908909; doi:10.1038/s41598-018-24565-y)
Supplement: Supplementary file 1 — Supplementary Figure [file 41598_2018_24565_MOESM1_ESM.pdf]

Aloperine Protects Mice against Bleomycin-induced Pulmonary Fibrosis by Attenuating Fibroblast Proliferation and Differentiation

Wanling Yin[1*] Jing Han[2*] Zhijun Zhang[3] Zaomu Han[1] Siyuan Wang[1#]

[1]Department of Gerontology, The Central Hospital of Wuhan, Tongji Medical College, Huazhong University of Science and Technology, Wuhan, China.

[2]Department of oncology, Tongji Hospital, Tongji Medical College, Huazhong University of Science and Technology, Wuhan, China.

[3]Reproductive medicine center, Taihe Hospital, Hubei University of Medicine, Shiyan, China.

*These authors contributed equally to this work

#Correspondence and reprint requests should be addressed to Mrs. Siyuan Wang (Tel: 86-27-6569-9792; email:wsy2012@whu.edu.cn), Department of Gerontology, The Central Hospital of Wuhan, Tongji Medical College, Huazhong University of Science and Technology, Wuhan, China.

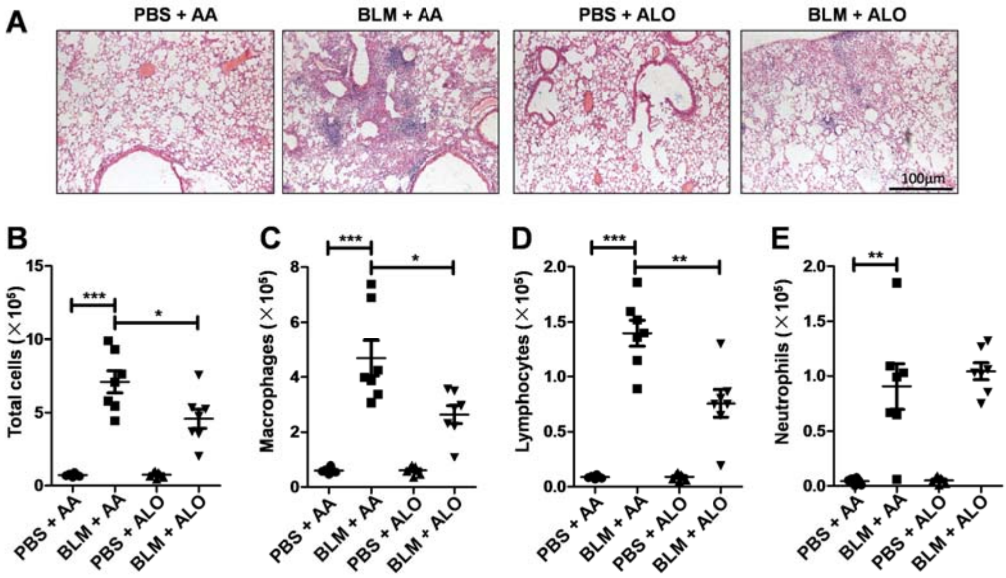

Supplementary Figure 1. Administration of aloperine attenuated lung inflammation after BLM induction. **A**: Representative results for H&E. Images were taken under ×200 amplification. **B:** Total number of inflammatory cells in BALF *samples*. **C-E:** Analysis of macrophages (**C**), lymphocytes (**D**), and neutrophils (**E**) in BALF samples. Six to 7 mice were included in each study group. Statistical analysis was performed by Tukey's test. *, $p < 0.05$; **, $p < 0.01$; ***, $p < 0.001$.

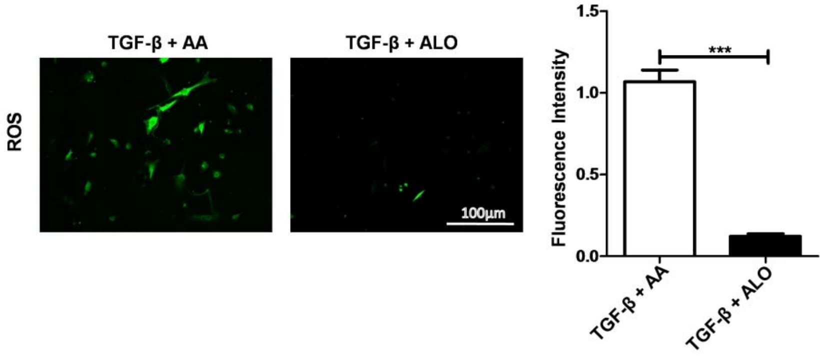

Supplementary Figure 2. Aloperine treatment inhibited fibroblast ROS production. Left panel: representative results for detection of DCFH-DA fluorescence in lung fibroblast following TGF-β stimulation. Right panel: quantitative mean fluorescence intensity of DCFH. Statistical analysis was performed by Mann-Whitney test. ***, $p < 0.001$.
